# Analysis of the Timing of Cervical Cerclage Treatment in Pregnant Women with Cervical Insufficiency and the Effect on Pregnancy Outcome

**DOI:** 10.1155/2022/8340009

**Published:** 2022-06-30

**Authors:** Deying He, Dan Zhao

**Affiliations:** Department of Obstetrics and Gynecology, Women and Children's Hospital of Chongqing Medical University, Chongqing 401120, China

## Abstract

**Purpose:**

To analyze the effect of the choice of timing of cervical cerclage treatment on pregnancy outcome in pregnant women with cervical insufficiency (CI).

**Methods:**

The case data of 160 pregnant women admitted to our hospital for cervical cerclage due to CI from January 2020 to September 2021 were sampled. They were divided into the early group (14∼18 weeks of pregnancy, *n* = 86), the middle group (19∼27 weeks of pregnancy, *n* = 74) according to the different gestational periods of surgical treatment, and into the elective group (elective operation, *n* = 71) and the emergency group (emergency operation, *n* = 89) according to the different timings of surgical treatment. To compare the pregnancy outcomes of the four groups and the effects of different treatment timings on pregnant women and newborns.

**Results:**

After the operation, the intrauterine infection rate in the early group was lower (8.14% (7/86)) than that (71.62% (53/74)) in the middle group, and the intrauterine infection rate (18.31% (13/71)) in the elective group was lower (61.80% (55/89)) than that in the emergency group (*P* < 0.05). After the operation, the late abortion rate in the early group was 8.14% (7/86) lower than 63.51% (47/74) in the middle group, and the late abortion rate in the elective group was 15.49% (11/71) lower than 61.80% (55/89) in the emergency group (*P* < 0.05). After the operation, the full-term birth rate (82.56% (71/86)) in the early group was higher (21.62% (16/74)) than that in the middle group, and the full-term birth rate (73.24% (52/71)) of the elective group was higher (24.72% (22/89)) than that in the emergency group (*P* < 0.05). After the operation, there was no significant difference in the preterm birth rate between the early group and the middle group (8.14% *vs* 14.86%), and between the elective group and the emergency group (11.27% *vs* 12.36%) (*P* > 0.05). There was no significant difference in neonatal Apgar scores between the early group and the middle group (7.30 ± 0.98 *vs* 7.14 ± 0.91) scores, and between the selective group and the emergency group (7.15 ± 0.82 *vs* 7.07 ± 1.07) scores (*P* > 0.05). There was no significant difference in gestational week extension time between the early group and the middle group (6.52 ± 1.77 *vs* 6.99 ± 1.69) days and between the elective group and the emergency group (6.44 ± 1.37 *vs* 6.82 ± 1.70) days (*P* > 0.05). The length of hospital stay was (7.28 ± 1.39 *vs* 10.89 ± 2.65) days in the early group and the middle group, with the early group being shorter than the middle group (*P* < 0.05), and the length of hospital stay was (8.72 ± 1.23 *vs* 9.30 ± 1.39) days in the elective group and the emergency group, with the elective group being shorter than the emergency group (*P* < 0.05).

**Conclusions:**

The therapeutic effect and pregnancy outcome of cervical cerclage are affected by the timing of treatment. Among them, the effect of elective operation at 14∼18 weeks of pregnancy is more ideal, which is worthy of clinical promotion.

## 1. Introduction

Cervical function is a physiological process that corresponds to the state of pregnancy. It is mainly manifested by the gradual softening of the cervix as the gestational weeks increase, the gradual shortening of the cervix as the fetus grows, and the gradual shortening and progressive dilatation of the cervix as contractions occur and intensify. The occurrence of cervical maturation processes that do not correspond to the current state of pregnancy, such as softening, shortening, or even dilatation of the cervix without a cause, can be considered the occurrence of cervical insufficiency (CI) [1]. Its role as an important component of the preterm birth syndrome is one of the main causes of miscarriage and preterm fetal birth in mid-to-late pregnancy [2–4]. The onset of CI is usually earlier than 24 weeks of gestation. It is mainly associated with various congenital factors (e.g., Mullerian duct malformation, fetal exposure to ethylene estradiol, cervical collagen, and elastin deficiency, etc.), acquired factors (e.g., rapid cervical dilatation or cervical laceration during delivery, scraping, and postcervical conization) or underlying factors (e.g., subclinical infection and local inflammation) leading to cervical dysfunction, incomplete atresia or flaccidity of the internal opening, and the inability of the cervix to support the growing fetus and amniotic fluid [5–7]. Clinically, 15% of recurrent spontaneous abortions (RSAs) in mid-pregnancy are associated with CI [8]. Some data [9] show that CI accounts for about 10% of the causes of preterm delivery, and the rate of preterm delivery in CI patients is more than three times that of non-CI patients. For this reason, it is crucial to improve pregnancy outcomes for pregnant women with CI. Cervical cerclage is currently the most effective and most commonly used treatment for CI. The shape and function of the cervical internal orifice of pregnant women are restored to normal through surgery, so that the tension of the cervical canal of pregnant women is enhanced, which can effectively prevent the extension and cervical dilatation of the lower segment of the uterus due to gravity. Thereby, the load on the lower uterine segment of the pregnant woman is reduced, which facilitates the prolongation of the gestational week and the increase of the full-term birth rate [10]. However, there are no standard definitions for its current choice of timing for surgical treatment. The clinical data of 160 pregnant women with CI were sampled in this study, with the aim of analyzing the effect of the choice of timing of treatment with cervical cerclage on pregnancy outcome in pregnant women with CI. See below for coverage.

## 2. Materials and Methods

### 2.1. Patients and Groups *P* > 0.05

The case data of 160 pregnant women admitted to our hospital for cervical cerclage due to CI from January 2020 to September 2021 were sampled. They were divided into the early group (14∼18 weeks of pregnancy, *n* = 86), the middle group (19∼27 weeks of pregnancy, *n* = 74) according to the different gestational periods of surgical treatment, and into the elective group (elective operation, *n* = 71) and the emergency group (emergency operation, *n* = 89) according to the different timing of surgical treatment. In terms of age, pregnancy time, spontaneous abortion history, preterm birth history, and other general information, the early group *vs* the middle group, the elective group *vs* the emergency group, none of the differences were statistically significant (*P* > 0.05), and were comparable (Table 1).

### 2.2. Diagnosis and Inclusion Criteria

The diagnostic criteria for CI in accordance with the guidelines of the ACOG [11] and in the context of our national situation: ① medical history: history of ≥2 painless mid-to late-term pregnancies with abortion or preterm birth, or history of cervical injury (surgical treatment for cervical lesions, etc. or history of cervical lacerations, etc.); ② vaginal examination: painless softening, shortening, and even dilatation of the cervical canal; ③ transvaginal sonography (TVS) showed shortening of the cervical canal <2.5 cm, separation of the endocervix, or a wedge-shaped or funnel-like change in the endocervix; ④ pre-pregnancy gynecological examination showed that the internal mouth of the cervix can reach the uterine cavity through No. 8 Hegar dilator; ⑤ non-pregnant hysterosalpingogram and hysteroscopy reveal a tubular enlargement of the funnel area in the isthmus of the uterus. The diagnosis is confirmed by meeting the first and any of the other 4 criteria above. In addition, in the absence of a previous history of multiple spontaneous abortions, or a history of only one abortion, the diagnosis can also be confirmed by the presence of painless cervical shortening and dilatation, a definite cervical length <2.5 cm, a CI suspected by ultrasound (cervical length <2.5 cm, cervical width >3.2 cm, cervical internal diameter >0.5 cm), or a high degree of suspicion of CI on examination (No. 8 uterine dilator can pass through the internal opening of the cervix). The medical records of the patients were complete; all of them were treated with cervical cerclage; and all of them voluntarily participated in the operation and signed the informed consent.

### 2.3. Exclusion Criteria

Exclusion Criteria were as follows: complicated with severe organic or systemic lesions; complicated with liver and kidney dysfunction or important organ injury; patients with mental diseases; complicated with coagulation dysfunction; contraindications or refusal of cervical cerclage; patients with reproductive tract infection; those with miscarriage or preterm birth due to other factors such as endocrine, infection, genetics, etc.; combined with ruptured membranes, placenta abruption; or severe congenital abnormalities requiring termination of the pregnancy.

### 2.4. Surgical Method

All patients were treated with cervical cerclage, which was performed as follows.

Before operation: magnesium sulfate injection (National Drug Certification H13022000, specification: 10 ml: 2.5 g) should be given one day before the operation; the first loading dose was 5 g, diluted to 100 ml with 5% dextrose injection, and then given intravenously rapidly within 30 minutes; after that 1-2 g per hour intravenously for maintenance, the total amount of 24 hours should not exceed 30 g. Dydrogesterone tablets (Imported Drug Registration No. H20130110, specification: 10 mg/tablet) were taken orally to reduce uterine sensitivity, 10 mg/time, q8h (once every 8 hours). If preoperative dilatation of the uterine orifice has already occurred, absolute bed rest in the head-low-hip-high position is required to reduce cervical pressure.

During operation: after the subarachnoid anesthesia took effect, the bladder truncation position was placed, and the perineum of the pregnant woman was fully exposed. First, the vulva and vagina were routinely disinfected, then the vaginal vault and cervix were fully exposed using a cervical forceps. To prevent injury to the fetal membranes, the operation should be performed gently. A Mersilene cervical band was used to perform the ring ligation, and afterwards, a “U” suture was applied. In cases where the amniotic sac had bulged into the cervical canal, the procedure was performed in a head-low-hip-high position, and the bulging amniotic sac had to be retracted before the procedure.

After operation: pregnant women were asked to rest in bed in the head-low-hip-high position after the operation, use of antibiotics for 1-2 days to prevent infection and maintain a clean vulva and use of magnesium sulfate injection 1-2 g/h for 2 days and dydrogesterone tablets 10 mg q8h orally for a week. Ultrasound and vaginal discharge were reviewed 1 week after the operation to check for ischemic necrosis of the cervical tissue and detachment of the annuloplasty thread. If there was no infection and no unstoppable contractions, the surgical sutures were removed at 37 weeks of gestation to avoid cervical laceration during delivery.

### 2.5. Evaluation Indexes

1. Comparison of pregnancy outcomes among the four groups of pregnant women: the evaluation indexes were intrauterine infection rate, late abortion rate, full-term birth rate, and preterm birth rate.
2. Comparison of the effects of different treatment timing on pregnant women and newborns: the evaluation indexes were neonatal Apgar score, gestational week extension time, and length of hospital stay.

### 2.6. Statistical Methods

Data processing was performed using the SPSS 22.0 software. Count data were expressed as percentages (%) and subjected to the *χ*^2^ test. The measurement data obeying normal distribution were expressed as mean ± standard deviation ($\bar{x}$ ±*s*) and subjected to *t*-test. The test level was *α* = 0.05, and *P* < 0.05 was considered a statistically significant difference.

## 3. Results

### 3.1. The Intrauterine Infection Rate of Early Group vs Middle Group and Elective Group vs Emergency Group

**Figure 1:**
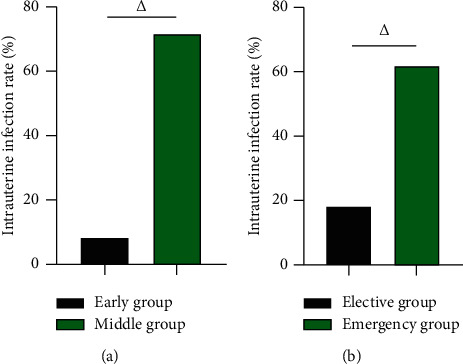
The intrauterine infection rate of early group vs middle group, elective group vs emergency group (%). (a) The intrauterine infection rate of early group vs middle group (%). (b) The intrauterine infection rate of elective group vs emergency group (%). △ was the comparison between groups, and the difference was statistically significant.

After the operation, the intrauterine infection rate in the early group was 8.14% (7/86) lower than 71.62% (53/74) in the middle group, and the intrauterine infection rate in the elective group was 18.31% (13/71) lower than 61.80% (55/89) in the emergency group (*P* < 0.05). In Figure 1.

### 3.2. The Late Abortion Rate of Early Group vs Middle Group and Elective Group vs Emergency Group

**Figure 2:**
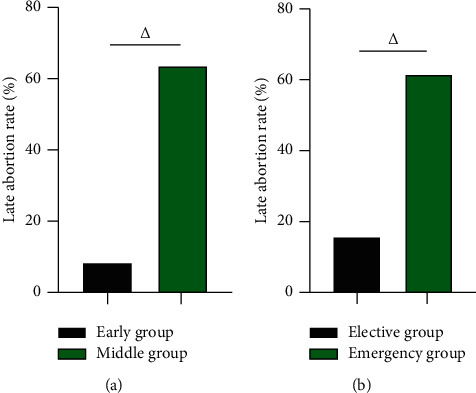
The late abortion rate of early group vs middle group, elective group vs emergency group (%). (a) The late abortion rate of early group vs middle group (%). (b) The late abortion rate of elective group vs emergency group (%). △ was the comparison between groups, and the difference was statistically significant.

After the operation, the late abortion rate in the early group was 8.14% (7/86) lower than 63.51% (47/74) in the middle group, and the late abortion rate in the elective group was 15.49% (11/71) lower than 61.80% (55/89) in the emergency group (*P* < 0.05) (Figure 2).

### 3.3. The Full-Term Birth Rate of Early Group vs Middle Group and Elective Group vs Emergency Group

**Figure 3:**
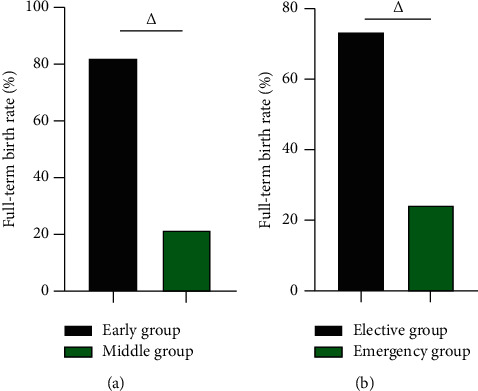
The full-term birth rate of early group vs middle group, elective group vs emergency group (%). (a) The full-term birth rate of early group vs middle group (%). (b) The full-term birth rate of elective group vs emergency group (%). △ was the comparison between groups, and the difference was statistically significant.

After the operation, the full-term birth rate in the early group was 82.56% (71/86) higher than 21.62% (16/74) in the middle group, and the full-term birth rate of the elective group was 73.24% (52/71) higher than 24.72% (22/89) in the emergency group (*P* < 0.05). In Figure 3.

### 3.4. The Preterm Birth Rate of Early Group vs Middle Group and Elective Group vs Emergency Group

**Figure 4:**
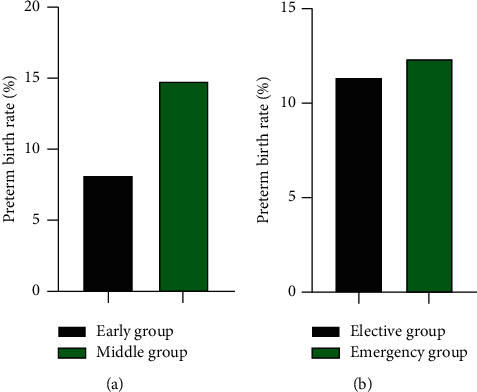
The preterm birth rate of early group vs middle group, elective group vs emergency group (%). (a) The preterm birth rate of early group vs middle group (%). (b) The preterm birth rate of elective group vs emergency group (%).

After the operation, there was no significant difference in the preterm birth rate between the early group and the middle group (8.14% *vs* 14.86%) scores, and between the elective group and the emergency group (11.27% vs 12.36%) scores (*P* > 0.05). In Figure 4.

### 3.5. The Neonatal Apgar Score of Early Group vs Middle Group and Elective Group vs Emergency Group

**Figure 5:**
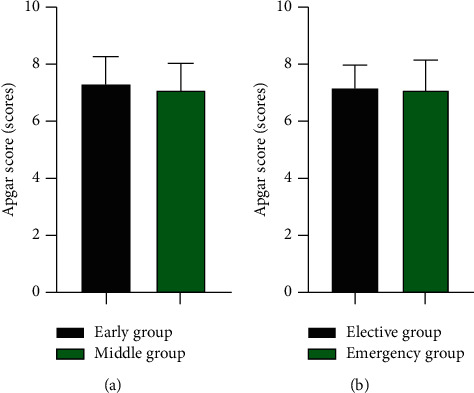
The neonatal Apgar score of early group vs middle group, elective group vs emergency group (scores). (a) The neonatal Apgar score of early group vs middle group (scores). (b) The neonatal Apgar score of elective group vs emergency group (scores).

There was no significant difference in neonatal Apgar score between the early group and the middle group (7.30 ± 0.98 *vs* 7.14 ± 0.91) and between the elective group and the emergency group (7.15 ± 0.82 *vs* 7.07 ± 1.07) (*P* > 0.05) (Figure 5).

### 3.6. The Gestational Week Extension Time of Early Group vs Middle Group and Elective Group vs Emergency Group

**Figure 6:**
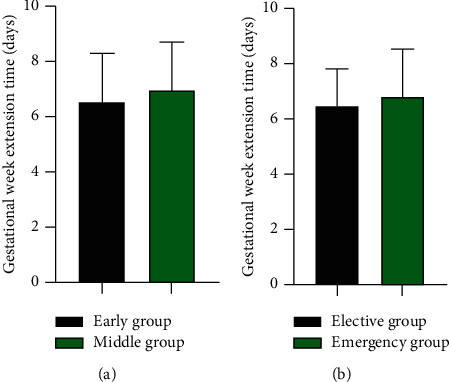
The gestational week extension time of early group vs middle group, elective group vs emergency group (days). (a) The gestational week extension time of early group vs middle group (days). (b) The gestational week extension time of elective group vs emergency group (days).

There was no significant difference in gestational week extension time between the early group and the middle group (6.52 ± 1.77 *vs* 6.99 ± 1.69) days and between the elective group and the emergency group (6.44 ± 1.37 *vs* 6.82 ± 1.70) days (*P* > 0.05) (Figure 6).

### 3.7. The Length of Hospital Stay of Early Group vs Middle Group and Elective Group vs Emergency Group

**Figure 7:**
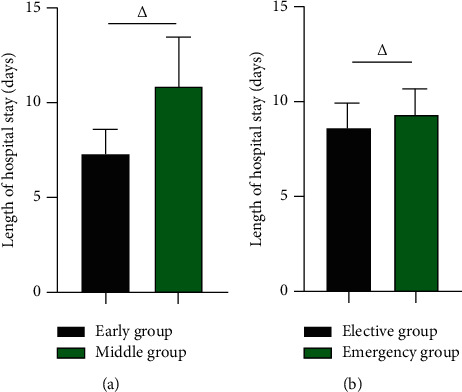
The length of hospital stay of early group vs middle group, elective group vs emergency group (days). (a) The length of hospital stay of early group vs middle group (days). (b) The length of hospital stay of elective group vs emergency group (days). △ was the comparison between groups, and the difference was statistically significant.

The length of hospital stay was 7.28 ± 1.39 vs 10.89 ± 2.65 days in the early group and in the middle group, with the early group being shorter than the middle group (*P* < 0.05), and the length of hospital stay was 8.72 ± 1.23 vs 9.30 ± 1.39 days in the elective group and the emergency group, with the elective group being shorter than the emergency group (*P* < 0.05). In Figure 7.

## 4. Discussion

CI can often lead to abortion or preterm birth of pregnant women. In the early stages of the disease, the internal orifice of the cervix can be shortened or funnel-shaped. Once premature delivery occurs, the organs of the newborn are not yet mature, and the survival probability is greatly reduced. If the anatomical structure of the cervix can be restored, it is expected to prolong the gestational week to the best gestational age and finally improve the perinatal outcome. Cervical cerclage is a common treatment for CI, which does not require any incision of the tissue during the procedure and therefore causes minimal damage to the surrounding tissue [12]. The principle of action is to narrow the endocervix by encircling the entire cervix with sutures, thus trimming the structure of the endocervix and strengthening the cervical canal tension as much as possible, preventing the extension of the lower uterine segment and the dilatation of the cervical opening, and assisting the endocervix to bear the gravity of the fetus and fetal appendages in the second trimester [13,14]. But the different gestational weeks and treatment timing of pregnant women will have different effects on the treatment effect.

It is generally accepted that if the gestational week is too early to exclude fetal abnormalities and because the placenta is not yet stable, surgery at this time can trigger abortion due to surgical stimulation [15]. If the gestational week is too late, the uterus is significantly enlarged, the uterine body rises into the abdominal cavity, and the cervix is elevated and shortened, which increases the risk of surgery and may cause premature rupture of membranes or contractions [16]. It has been reported [17] that the use of prophylactic cervical cerclage (i.e., treatment with cervical cerclage at 14 to 18 weeks of pregnancy) significantly improves the success rate of the procedure and can effectively reduce intraoperative bleeding and shorten the length of hospital stay. Using this as the cut-off value, the early group was drawn from 14 to 18 weeks of gestation, and the middle group was drawn from 19 to 27 weeks of gestation in this study.

The results showed that after the operation, the intrauterine infection rate and late abortion rate were lower in the early group than in the middle group, the full-term birth rate was higher in the early group than in the middle group, and the length of hospital stay was shorter in the early group than with the middle group (*P* < 0.05); after surgery, the intrauterine infection rate and late abortion rate were lower in the elective group than in the emergency group, the full-term birth rate was higher in the elective group than in the emergency group, and the length of hospital stay was shorter in the elective group than with the emergency group (*P* < 0.05). This suggests that elective cervical cerclage at 14 to 18 weeks of gestation in patients with CI will help reduce the rate of intrauterine infection and late miscarriage, increase the rate of full-term productivity, and improve overall pregnancy outcome due to rapid postoperative recovery. Conversely, if the disease is diagnosed between 19 and 27 weeks of gestation, emergency surgery should be performed as soon as possible, with strict postoperative monitoring and control to prevent infection and ensure a good pregnancy outcome. The reason why there are more adverse pregnancy outcomes with emergency cervical cerclage at 19 to 27 weeks of gestation compared to elective cervical cerclage at 14 to 18 weeks of gestation may be due to the fact that as the gestational age increases, the pressure in the pregnant woman's uterus gradually decreases and the cervical opening gradually dilates. This may be due to the fact that as the gestational age increases, the pressure in the pregnant woman's uterus gradually decreases and the cervical opening gradually dilates. At this time, if the operation is performed too late or too urgently, it may be more difficult and less effective due to a series of complications such as dilatation of the cervical opening, bulging of the fetal sac, and shortening/disappearance of the cervical canal. For example, pregnant women may develop intrauterine fetal infections due to the reduced ability of the cervical mucus plug to block bacterial invasion [18]. The dilatation of the cervical opening and the bulging of the maternal amniotic sac out of the vagina, which requires repeated upward pushing and retraction of the bulging amniotic sac during surgery, may increase the risk of rupture of the fetal membranes and cause intrauterine infection [19]. Also, in patients with dilatation of the endocervix already occurring, the height of the ring ligation makes it difficult to reach the level of the endocervix, so it is not effective in maintaining the length of the cervix and the support it provides to the cervix, and after surgery, as the gestational week increases and the pregnant woman's uterus increases, it is highly likely that the pregnancy will have to be terminated due to the increased force of cervical dilatation, so the full-term productivity is low [20]. The above problems not only affect the surgical effect of patients but also lead to the corresponding extension of hospital stay, which is not conducive to the postoperative recovery of CI patients and the maintenance of good pregnancy outcomes.

The results of this study also showed that after operation, there was no statistical significance in the preterm birth rate, neonatal Apgar score, and gestational week prolongation time in the early group compared with the middle group, and in the elective group compared with the emergency group (*P* > 0.05). This may be because compared with other treatment schemes, cervical cerclage itself has the characteristics of short operation time, no trauma, simple operation, fast postoperative recovery, and good effect [21]. And whether it is 14 to 18 weeks of gestation or 19 to 27 weeks of gestation, whether it is an elective operation or an emergency operation, all four can eventually improve the cervical structure and physiological function of CI patients effectively, and together with the later treatment of antiinflammation and suppression of contractions, the patients can eventually prolong the pregnancy, even deliver successfully, etc. [22–24].

To summarize, the outcome of cervical cerclage in the treatment of pregnant women with CI in mid-pregnancy and pregnancy outcomes are influenced by the timing of treatment. Among them, elective surgical intervention at 14–18 weeks of gestation is more ideal, which can effectively prevent intrauterine infection and late abortion in pregnant women, and the patient recovers quickly after surgery and the hospital stay is greatly shortened, which is a reliable method to improve pregnancy outcome and quality of life in patients treated with cervical cerclage in CI and is worthy of clinical promotion. However, if the diagnosis of CI is not confirmed until 19∼27 weeks of pregnancy, emergency surgical treatment should be taken immediately, and postoperative monitoring and infection control should be strictly carried out to ensure a good pregnancy outcome for pregnant women.

## Figures and Tables

**Table 1:** Comparison of general information of pregnant women in each group.

Group	Age (years)	Pregnancy time (times)	Spontaneous abortion history (cases)			Preterm birth history (cases)
			Once	Twice	3 times	
Early group (*n* = 86)	30.78 ± 3.00	3.14 ± 0.38	46	29	11	6
Middle group (*n* = 74)	29.97 ± 2.23	3.12 ± 0.52	40	25	9	7
*t*/*χ*^2^	1.619	0.280	0.005	0.001	0.014	0.328
*P*	0.107	0.780	0.943	0.993	0.905	0.567
Elective group (*n* = 71)	31.10 ± 2.17	3.11 ± 0.36	42	24	6	5
Emergency group (*n* = 89)	30.40 ± 2.39	3.15 ± 0.51	44	30	14	8
*t*/*χ*^2^	1.659	0.579	1.500	0.001	1.914	1.330
*P*	0.099	0.564	0.221	0.990	0.167	0.249

## Data Availability

The data used or analyzed in the current study are available from the associated author.
